# Diffusion Tensor Imaging for In Vivo Detection of Degenerated Optic Radiation

**DOI:** 10.5402/2011/648450

**Published:** 2011-12-27

**Authors:** Georg Michelson, Tobias Engelhorn, Simone Waerntges, Arnd Doerfler

**Affiliations:** ^1^Department of Opthtalmology, University Erlangen-Nuremberg, Schwabachanlage 6, 91054 Erlangen, Germany; ^2^Department of Neuroradiology, University Erlangen-Nuremberg, Schwabachanlage 6, 91054 Erlangen, Germany

## Abstract

Glaucomatous optic nerve atrophy may continue to the linked optic radiation by transneuronal degeneration, as described in animal models of glaucoma. In vivo visualization of the visual pathway represents a new challenge in the field of ophthalmology. We present a new approach for illustration of the optic radiation by diffusion tensor imaging (DTI) based on magnetic resonance imaging (MRI). The DTI was established by use of a 3T high-field scanner. The case of a patient with primary open-angle glaucoma is opposed to this one of a healthy subject to demonstrate the visible rarefication of the optic radiation. The goal was to introduce the technique of the DTI also in ophthalmology and to demonstrate that it may be useful to judge glaucoma-related differences.

## 1. Introduction

Transneuronal degeneration is a process of primary neuron injury affecting the linked distal neurons. It was described for pathophysiological changes in neurological diseases such as Alzheimer's disease [1] and brain trauma [2]. More recent studies have suggested that this damage also occurs in the development of glaucoma [3–5]. Loss of retinal ganglion cells in the retina and their axons that represent the optic nerve (3rd neuron) [6] and a loss of astrocytes [7] is the predominant finding in primary open-angle glaucoma. The axons of various retinal ganglion cell subtypes, differing in specific morphology and function, exit the eye ball and finally converge to anatomically distinguishable layers of the lateral geniculate nucleus (LGN) [8] where a loss of neural cells has also been described in glaucoma [9]. The LGN serves as a “relay station” that transmits the information via the 4th neuron to the primary visual cortex (V1) [9]. Even in V1 neurons were found to be reduced in glaucomatous optic nerve atrophy [6]. The loss of axons of the 3rd neuron and astrocytes, which are placed between the optic nerve fibers becomes visible by a “cupping” of the optic nerve head [10]. Functionally, glaucoma results in a loss of visual function, which is detectable by determination of the visual field (white-white perimetry) and of the spatial-temporal contrast sensitivity (frequency doubling test, FDT) [11, 12].

It is not clear if the loss of axons of the 3rd neuron causes directly an injury of the 4th neuron in humans. However, animal experiments recently have shown that glaucomatous loss of axons of the 3rd neuron is followed by a loss of the LGN volume which indicates a reduced number of 4th neurons [9]. Furthermore, with increasing loss of retinal ganglion cells an increasing loss in LGN neurons was described [9].

In vivo detection of glaucomatous changes along the visual pathway and its conjunction to intraocular findings represents a special challenge. We report a new approach for in vivo visualization of pathological changes of the optic radiation (4th neuron) in glaucoma by use of diffusion tensor imaging (DTI). DTI is based on the random motion of water molecules, which is associated with their thermal energy at body temperature (Brownian motion) and which is known as “diffusion” [13]. In the presence of a strong magnetic gradient a loss of the magnetic resonance signal results as a consequence of the dephasing of spin coherence [14]. In each voxel the eigenvectors can be characterized from the generated diffusion-weighted magnetic resonance images [14]. In white matter consisting of axons in all directions of a three-dimensional space the diffusion of free water molecules is different (anisotropy) [15, 16]. Predominantly the orientation of fiber tracts and their micro- and macrostructural features influences the diffusion anisotropy [17] and provides the direction of the largest eigenvector. Macroscopically the degree of anisotropy assigned to a definite voxel is affected by the variability in the orientation of all white matter tracts in this imaging voxel [17]. Even reconstructions of fiber crossing and branching within the optic chiasm and the LGN are possible by DTI. Thus, the entire visual pathway from the optic nerve to the visual cortex may be reconstructed [18].

In our work we intended to introduce the DTI as a suitable examination method to detect changes of the optic radiation in glaucoma patients.

## 2. Materials and Methods

A case report in comparison to a healthy age-matched control subject is shown. Subjects received a questionnaire requesting age, gender, known cardiovascular risk factors (i.e., arterial hypertension, diabetes, smoking history), and cardiovascular events (i.e., myocardial infarction, peripheral arterial disease, transient ischemic attack, and stroke). Eyes were assessed by a full ophthalmological examination with dilated pupils and judgement of automated perimetry (Octo 101 dG2, Interzeag, Schlieren, Switzerland), of spatial-temporal contrast sensitivity (FDT, frequency doubling test, Carl Zeiss Meditec AG, Jena, Germany), and nonmydriatic fundus images (KOWA, Nonmyd-alpha 45, Japan).

MRI was performed on a 3T high-field scanner (Magnetom Tim Trio, Siemens, Erlangen, Germany) with a gradient field strength up to 45 mT/m (72 mT/m effective). The anatomical data were obtained in a T1-weighted 3D-MPRAGE sequence (TR = 900 ms, TE = 3 ms, FoV = 23 × 23 cm, acquisition matrix size = 512 × 256 reconstructed to 512 × 512, reconstructed axial plans with 1.2 mm slice thickness). For detection of microangiopathy, a heavily T2-weighted fluid-attenuated inversion recovery (FLAIR) sequence covering the whole brain was acquired (TR = 10000 ms, TE = 115 ms, matrix size = 512 × 512). DTI was performed in the axial plane with 4 mm slice thickness and a 1 mm interslice separation using a single-shot, spin echo, echo planar imaging (EPI) diffusion tensor sequence thus covering the whole visual pathway (TR = 3400 ms, TE = 93 ms, FoV = 23 × 23 cm, acquisition matrix size = 128 × 128 reconstructed to 256 × 256, number of signal averages = 7, partial Fourier acquisition = 60%). Diffusion weighting with a maximal b-factor of 1000 s/mm^2^ was carried out along 15 icosahedral directions complemented by one scan with *b* = 0. Datasets were automatically corrected for imaging distortions and coregistered in reference to T1-weighted MPRAGE images. These and further calculations such as determining the independent elements of the diffusion tensor, deriving the corresponding eigenvalues and eigenvectors, were performed with a dedicated software package (Neuro 3D, Siemens, Erlangen, Germany).

The study was conducted in accordance with the Declaration of Helsinki on Biomedical Research Involving Human Subjects. Written informed consent was obtained from all subjects. 

## 3. Results and Discussion

### 3.1. Reliability of Semiautomated Segmentation of the Optic Radiation

The images of 14 patients were tested for the reliability of the corrected optic radiation segmentation. Two observers postprocessed manually the automatically provided segmentation to exclude cerebral structures that are not a part of the optic radiation and the fractional anisotropy was calculated. Cronbach-*α* at the 95% confidence interval for FA was 0.990 (right optic radiation, *F* = 2.340, *P* = 0.150) and 0.886 (left optic radiation, *F* = 1.086, *P* = 0.316) with df1 = 1 and df2 = 13. Thus, the intraclass correlation coefficients of reliability of the semiautomated segmentation were within a very good range of reliability.

**Figure 1:**
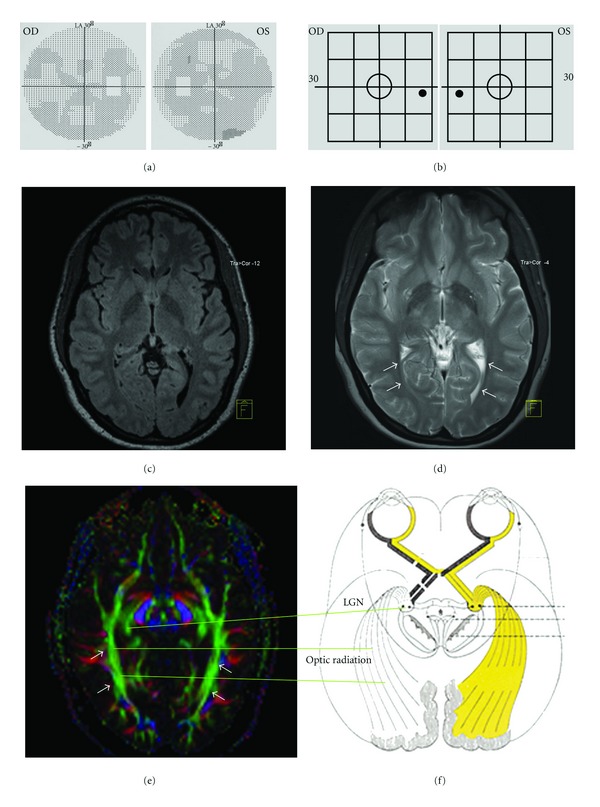
DTI of the optic system in a healthy subject. (a) The automated perimeter did no show visual field defects. (b) The frequency doubling test likewise did not show any impaired spatial-temporal contrast sensitivity. (c) The findings in the T2-weighted MRI were normal. (d) The localization of the optic radiations (arrows) is labelled in the T1-weighted MRI. (e) DTI reveals that the optic radiation in the occipital lobe (arrows) is developed vigorously and completely. (f) The schematic drawing shows the anatomy of the visual pathway, particularly of the optic radiation and lateral geniculate nucleus (LGN).

**Figure 2:**
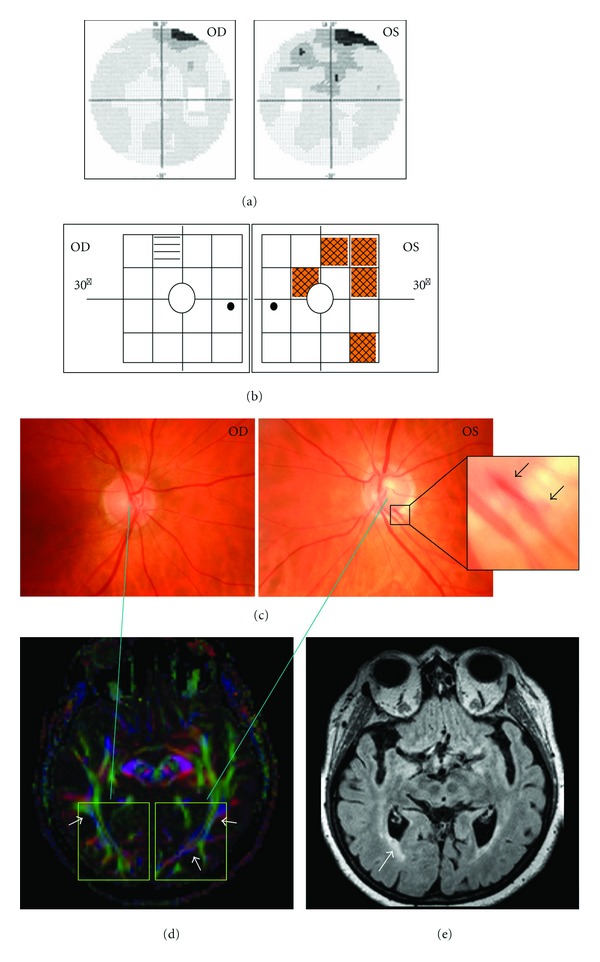
Glaucomatous optic nerve atrophy (case no. 1). Woman, 68 yr, OD/OS with primary open-angle glaucoma, in OS a parapapillary bleeding of the optic nerve head; topical medication: brimonidine, *latanoprost*, dorzolamide. (a) In both eyes (OS (left) > OD [right]) the automated perimeter showed predominantly superior visual field defects due to a loss of axons of the 3rd neuron. (b) The frequency doubling test indicated impaired spatial-temporal contrast sensitivity primarily in OS in the superior and temporal area as well as nasal near the center. (c) Typical signs of glaucomatous optic nerve atrophy were recorded by a nonmydriatic fundus camera that is in OS a small rim area, smaller inferior rim than temporal and a parapapillary bleeding (arrows). (d) DTI reveals significant rarefication of the optic radiation (arrows) in both occipital lobes (left > right). (e) Circumscribed microangiopathy (arrow) was diagnosed in the optic radiation based on MRI (FLAIR sequence).

Compared to the optic radiation of a healthy volunteer the DTI of a patient with primary open-angle glaucoma appears rarefied (Figures 1 and 2). The number of voxels in the optic radiation of the POAG patient is on average a third smaller than in the control subject (Table 1). Calculations of the number of voxels in the manually segmented optic radiation have confirmed the rarefied optic radiation in glaucoma patients [19]. The underlying pathophysiology of the glaucoma disease is based on a damage of the 3rd neuron with loss of function. It is discussed that a “transneuronal degeneration” to the 4th neuron proceeds. Most studies examined primate models of experimentally induced glaucoma. It was reported that in unilateral experimental glaucoma in addition to the damage of retinal ganglion cells also the post-retinal magno- and parvocellular layers of the LGN of the thalamus [3] and the primary visual cortex may be impaired [9]. Transneuronal degeneration was also found in a postmortem human study of the optic nerve, LGN, and visual cortex [7]. In a primate model of glaucoma the neuronal degeneration was shown to proceed from the LGN to the visual cortex [20, 21]. It is progressive with increasing nerve fiber loss of the 3rd neuron [9]. A loss of 50–60% of the ganglion cells may be already present if visual field defects are detectable by perimetry [22]. It was also shown in experimental glaucoma that some of the 4th neurons in the LGN have died and surviving neurons were atrophic [3–5]. Because impairment of the visual field is associated with optic nerve degeneration a certain probability for neurodegeneration of the 4th neuron of the visual pathway may be adopted and even was confirmed.

## 4. Conclusions

This case report demonstrates that the DTI allows an in vivo visualization of the posterior part of the visual pathway. This new technique in ophthalmology may be applicable for earlier diagnosis of glaucomatous changes and for noninvasive therapy monitoring in the future. However, presently this method is relatively expensive. Additionally, the impact for the different entities of optic nerve atrophy should be clearly validated prior to implementation into routine diagnostic.

## Figures and Tables

**Table 1:** Characteristics of the case reports.

	Case		Control	
ID	HS		JS	
Age [yr]	68		25	
Gender	F		F	
Concomitant diseases	HT, HC stroke 12 yr ago		Crohn's disease	
DTI diagnosis of optic radiation	Rarefication L > R		Vigorously and completely developed	
MRI diagnosis	Moderate cerebral microangiopathy, level 2		No cerebral microangiopathy	

	OD	OS	OD	OS

Eye diagnosis	POAG	POAG, parapapillary bleeding	Vital papilla	Vital papilla
Visual acuity	1.0	0.6	1.25	1.0
IOP [mm Hg](Case with local therapy)	18	17	14	11
Octopus MD [dB]	3.6	6.6	−0.6	0.3
FDT (≤50 sec)	42	74	36	34
HRT Disc area (1.69–2.82 mm^2^)	1.855	1.718		
HRT Cup area (0.26–1.27 mm^2^)	0.269	0.166		
HRT Rim area (1.20–1.78 mm^2^)	1.586	1.552		

	Right OR	Left OR	Right OR	Left OR

DTI Voxel No.	575	470	848	717

F: female; OD: right eye; OS: left eye; OR: optic radiation; POAG: primary open-angle glaucoma; IOP: intraocular pressure, MD: mean defect; FDT: frequency doubling test (loss of spatial-temporal contrast sensitivity: A = mild relative >95%, B = moderate relative >98%, C = severe >99%; number of fields of a maximum of 17); HRT: Heidelberg Retina Tomograph; DTI: diffusion tractography imaging; HT: arterial hypertension; HC: hypercholesterolemia.
